# Mortality, functional outcome, and bleeding risk after early versus delayed thrombectomy

**DOI:** 10.1007/s11357-025-01915-z

**Published:** 2025-10-16

**Authors:** Zoltan Ungvari, János Tibor Fekete, Mónika Fekete, Andrea Lehoczki, Rafal Gulej, Farzaneh Sorond, Eric Liotta, Calin I. Prodan, Peter Toth, Csaba Kiss, Anna Ungvari, Balázs Győrffy

**Affiliations:** 1 Vascular Cognitive Impairment, Neurodegeneration and Healthy Brain Aging Program, Department of Neurosurgery, University of Oklahoma Health Sciences Center, Oklahoma City, OK USA; 2 Stephenson Cancer Center, University of Oklahoma, Oklahoma City, OK USA; 3 Oklahoma Center for Geroscience and Healthy Brain Aging, University of Oklahoma Health Sciences Center, Oklahoma City, OK USA; 4 Department of Health Promotion Sciences, College of Public Health, University of Oklahoma Health Sciences Center, Oklahoma City, OK USA; 5 International Training Program in Geroscience, Doctoral College, Health Sciences Division/Institute of Preventive Medicine and Public Health, Semmelweis University, Budapest, Hungary; 6 Dept. of Bioinformatics, Semmelweis University, Budapest, 1094 Hungary; 7 Cancer Biomarker Research Group, Institute of Molecular Life Sciences, HUN-REN Research Centre for Natural Sciences, Budapest, 1117 Hungary; 8 Institute of Preventive Medicine and Public Health, Semmelweis University, Budapest, Hungary; 9 Fodor Center for Prevention and Healthy Aging, Semmelweis University, Budapest, Hungary; 10 Doctoral College, Health Sciences Division, Semmelweis University, Budapest, Hungary; 11 Department of Neurology, Northwestern University, Feinberg School of Medicine, Chicago, USA; 12 Veterans Affairs Medical Center, Oklahoma City, OK USA; 13 Department of Neurology, University of Oklahoma Health Sciences Center, Oklahoma City, OK USA; 14 Department of Neurosurgery, Medical School, University of Pecs, Pecs, Hungary; 15 Dept. of Biophysics, Medical School, University of Pecs, Pecs, 7624 Hungary

**Keywords:** Acute ischemic stroke, Large vessel occlusion, Endovascular thrombectomy, Treatment delay, Late intervention, Functional outcome, Mortality, Symptomatic intracranial hemorrhage, Meta-analysis, Time-to-treatment, Reperfusion therapy, Stroke outcomes

## Abstract

Endovascular thrombectomy (EVT) is an established treatment for acute ischemic stroke, due to large vessel occlusion (LVO), but the optimal time window for intervention remains a subject of ongoing debate. We aimed to assess the impact of treatment timing on mortality, functional outcomes, and safety by comparing early (≤ 6 h) versus late (> 6–24 h) EVT. We conducted a systematic review and meta-analysis to evaluate the effect of time to intervention on outcomes of endovascular thrombectomy (EVT) in acute ischemic stroke. Four databases (PubMed, Web of Science, Cochrane Library, and EMBASE) were searched for studies published between 2000 and 2024. Eligible randomized controlled trials and cohort studies reported on 90-day mortality, functional outcome (modified Rankin Scale, mRS), or symptomatic intracranial hemorrhage (sICH), stratified by treatment timing (≤ 6 h vs. > 6–24 h from symptom onset). Pooled incidence rates, incidence rate differences (IRD), and incidence rate ratios (IRR) were calculated using random-effects models. Eighteen studies met inclusion criteria. The pooled incidence of symptomatic intracranial hemorrhage (sICH) was 0.19 events per person-year (95% CI: 0.12–0.26) in the early group and 0.23 events per person-year (95% CI: 0.11–0.35) in the late group, with no significant difference between groups (incidence rate difference [IRD] − 0.028; *p* = 0.33, incidence rate ratio [IRR] 0.88; *p* = 0.33). For mortality, early EVT showed a significantly lower incidence rate of 0.66 events per person-year (95% CI: 0.51–0.82) compared to 0.77 events per person-year (95% CI: 0.63–0.91) in the late EVT group (IRD − 0.148; *p* = 0.0012, IRR 0.81; *p* = 0.0014). Functional independence was more frequent in the early group (1.72; 95% CI: 1.42–2.01) than in the late group (1.45; 95% CI: 0.91–1.98) (IRD 0.32; *p* < 0.0001, IRR 1.22; *p* < 0.0001). Heterogeneity was moderate to high across outcomes. The timing of endovascular thrombectomy significantly influences clinical outcomes in acute ischemic stroke. Our analysis shows that early intervention (within 6 h) is associated with a significantly lower mortality rate and a higher likelihood of achieving functional independence at 90 days compared to late intervention (beyond 6 up to 24 h). The incidence of symptomatic intracranial hemorrhage did not differ significantly between the groups, suggesting that late treatment does not increase safety risks. These findings underscore the importance of minimizing treatment delays, while also supporting the continued use of EVT in selected patients beyond the 6-h window.

## Introduction

Acute ischemic stroke (AIS) is a leading cause of death and long-term disability worldwide, posing a substantial burden on healthcare systems and society at large [1–3]. The advent of endovascular thrombectomy (EVT) has revolutionized the treatment of AIS due to large vessel occlusion (LVO), offering significant improvements in clinical outcomes compared to intravenous thrombolysis alone [4, 5]. EVT is now a cornerstone of AIS management, particularly for patients presenting within a defined time window from symptom onset.

While the efficacy of EVT is well established, the optimal timing of intervention remains an area of active investigation [6]. Optimal neurological recovery is achieved when reperfusion is initiated as early as possible—ideally within the so-called golden hour after onset—underscoring that even within accepted therapeutic windows, earlier intervention generally yields superior outcomes. Landmark trials have demonstrated the benefit of EVT within 6 h of symptom onset, and subsequent studies have extended the treatment window to 24 h in selected patients using advanced imaging criteria [7–9]. However, questions remain regarding how outcomes differ between early (≤ 6 h) and delayed (> 6–24 h) intervention, especially in real-world settings where imaging availability and patient selection strategies vary.

The relationship between treatment delay and outcomes is biologically plausible, as neuronal death progresses rapidly in the ischemic core [10, 11]. Delayed reperfusion may result in reduced salvageable tissue and higher risk of complications, including hemorrhagic transformation [10, 12, 13]. However, emerging data suggest that some patients with acute ischemic stroke exhibit relatively slow progression of infarct expansion, maintaining viable penumbral tissue well beyond conventional time windows [14–18]. This evolving understanding has shifted the focus from rigid time-based treatment thresholds toward individualized assessment based on biological and imaging markers of infarct progression [19, 20]. One possible explanation for this variability is genetic heterogeneity in collateral circulation, which influences the degree of cerebral perfusion and the survival of at-risk tissue. Despite these advances, the overall safety and efficacy of EVT beyond 6 h remain incompletely characterized across diverse clinical scenarios.

To address this knowledge gap, we conducted a systematic review and meta-analysis of studies reporting mortality, functional outcomes, and bleeding risk after early versus delayed EVT. By synthesizing evidence from both randomized controlled trials and high-quality observational studies, we aimed to clarify the impact of treatment timing on key clinical endpoints. Our findings may inform clinical decision-making, refine treatment guidelines, and support efforts to minimize delays in reperfusion therapy.

## Methods

### Study design and data sources

We conducted a systematic literature review and meta-analysis to evaluate the effect of delayed endovascular thrombectomy (EVT) on mortality and functional outcomes among patients with acute ischemic stroke. Our goal was to pool data from high-quality randomized controlled trials (RCTs) and large cohort studies to better understand the impact of treatment timing on patient prognosis.

For this review, “early” EVT was defined as ≤ 6 h from last known well (LKW) or symptom onset, and “late” EVT as > 6 to 24 h from LKW, consistent with DAWN and DEFUSE-3 criteria. When studies reported door-to-groin or imaging-to-puncture times, we converted to LKW-based intervals when feasible.

We began by systematically searching four major databases: PubMed, Web of Science, the Cochrane Library, and EMBASE. The search strategy combined a set of predefined keywords, including “endovascular thrombectomy delay stroke mortality,” “time to treatment ischemic stroke thrombectomy,” “thrombectomy outcomes based on time window,” “functional outcomes thrombectomy stroke,” and “odds ratio thrombectomy stroke mortality.” To ensure consistency and relevance, we limited our search to full-text, peer-reviewed articles published between 2000 and 2024. Finally, the reference lists of identified publications and other similar meta-analyses and reviews were also utilized to gather additional data [8, 21–27]. Most included studies used modern stent retriever or aspiration systems, improving applicability to contemporary EVT practice.

### Inclusion and exclusion criteria

We included studies that met the following criteria: a confirmed diagnosis of acute ischemic stroke, use of EVT as the primary intervention, and assessment of treatment timing in relation to clinical outcomes. Eligible studies reported on either 90-day mortality or functional outcome using the modified Rankin Scale (mRS). Only RCTs or large-scale cohort studies with adequate sample sizes and methodological rigor were considered. To ensure population consistency, we primarily included studies that focused on large vessel occlusions of the anterior circulation, predominantly involving the intracranial internal carotid artery (ICA) and proximal segments of the middle cerebral artery (MCA), with or without inclusion of dominant M2 branches. We excluded studies conducted on animal models, investigations that lacked sufficient follow-up data, and those focusing solely on pharmacologic or non-endovascular interventions. All extracted data were independently verified by multiple reviewers to ensure consistency and accuracy. Discrepancies were resolved through consensus.

### Data extraction and variables

After identifying eligible studies, we extracted relevant data in a standardized fashion. From each study, we recorded the time to intervention, categorized by treatment windows, along with details on the efficacy of EVT relative to time delay. We also documented methodological characteristics including study design, inclusion and exclusion criteria, and treatment arms. All included trials compared EVT—using mechanical devices such as stent retrievers, aspiration systems, or second-generation thrombus retrieval tools—against control groups receiving medical therapy alone. Specific device types and generations were not consistently reported across studies; therefore, formal adjustment for device selection was not feasible. However, based on the time period of the included studies and their alignment with current guideline-supported practice, it is reasonable to assume that the majority utilized later-generation thrombectomy devices, similar to those used in the pivotal trials establishing EVT efficacy within the 6-h window.

### Outcome analysis

We categorized interventions into early and late treatment windows, with early defined as within 6 h from symptom onset or last known well, and late as beyond 6 up to 24 h from symptom onset or last known well. Our primary outcomes included 90-day mortality and functional status, as measured by the modified Rankin Scale (mRS), where a score of 0 to 2 denoted functional independence. For safety outcomes, we focused on the incidence of symptomatic intracranial hemorrhage (sICH) as a major complication of the procedure. ICH was most often defined according to ECASS II or III criteria, with the remainder using SITS-MOST or NINDS definitions. Variability in sICH definitions is acknowledged as a source of potential heterogeneity.

### Data preparation

To quantify event frequency across studies, we first estimated the total number of patient-years for each outcome domain. This was calculated by multiplying the number of patients (*N*) in each study by an assumed follow-up duration of 90 days, then converting the results into years using the following formula: person-time = (*N* × 90)/365.25. The assumption of a 90-day follow-up period was applied consistently across all studies to enable standardized estimation of person-time, recognizing this reflects the typical follow-up timeframe used in stroke outcome research.

### Meta-analysis methods

Subsequent statistical analyses were conducted using the web-based platform available at https://metaanalysisonline.com[28]. For the three outcomes—intracranial hemorrhage (ICH), mortality, and functional outcome based on the modified Rankin Scale (mRS)—we calculated pooled incidence rates separately for two predefined groups based on treatment timing: an early treatment group, comprising patients who received EVT within 6 h, and a late treatment group, comprising patients treated between 6 and 24 h. Pooled estimates were calculated using a random-effects model with the inverse variance method. No transformation was applied to the incidence rates; results are reported as untransformed pooled rates.

Following the calculation of pooled incidence rates within each group, we directly compared these rates between groups by calculating both the incidence rate ratio (IRR) and the incidence rate difference (IRD). IRRs were estimated using Poisson-based models with logarithmic transformation, and Wald-type *Z* tests were used to derive 95% confidence intervals and *p*-values. IRDs were evaluated using normal approximation, also with confidence intervals and statistical significance testing.

### Graphical representation of the results

Forest plots were generated to visualize the study-level and pooled incidence rates within each treatment group, while funnel plots were used to explore the potential presence of publication bias. Heterogeneity across studies was quantified using the *I*^2^ statistic, which describes the proportion of total variation attributable to between-study heterogeneity rather than chance.

## Results

**Fig. 1:**
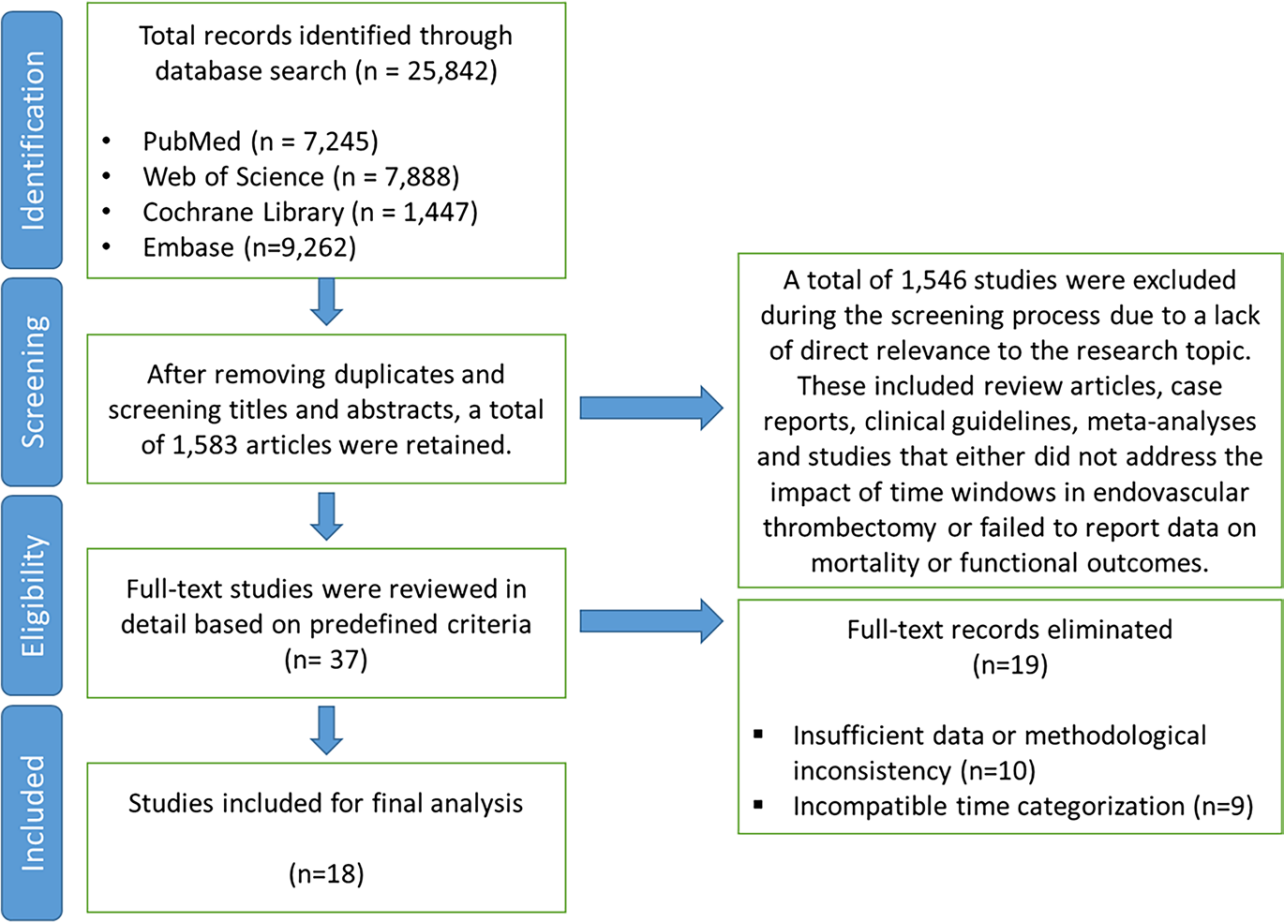
PRISMA flow diagram depicting the study selection process. A total of 25,842 records were identified through systematic database searches across four major sources: PubMed, Web of Science, Cochrane Library, and Embase. After removing duplicates and screening titles and abstracts, 1583 records remained. Following full-text assessment of 37 studies based on predefined eligibility criteria, 19 studies were excluded due to insufficient data, methodological inconsistencies (*n* = 10), or incompatible time categorization (*n* = 9). Ultimately, 18 studies were included in the final meta-analysis evaluating the impact of early (≤ 6 h) versus late (> 6–24 h) endovascular thrombectomy on mortality, functional outcomes, and symptomatic intracranial hemorrhage in acute ischemic stroke

Out of the 37 full-text studies, 10 were excluded due to insufficient data availability or methodological inconsistencies. An additional 9 studies were excluded because treatment timing was not reported in a format compatible with the predefined categories of ≤ 6 h and > 6 to 24 h. A total of 18 relevant studies were included in this meta-analysis (Fig. 1) [29–46].

### Symptomatic intracranial hemorrhage (sICH)

**Fig. 2:**
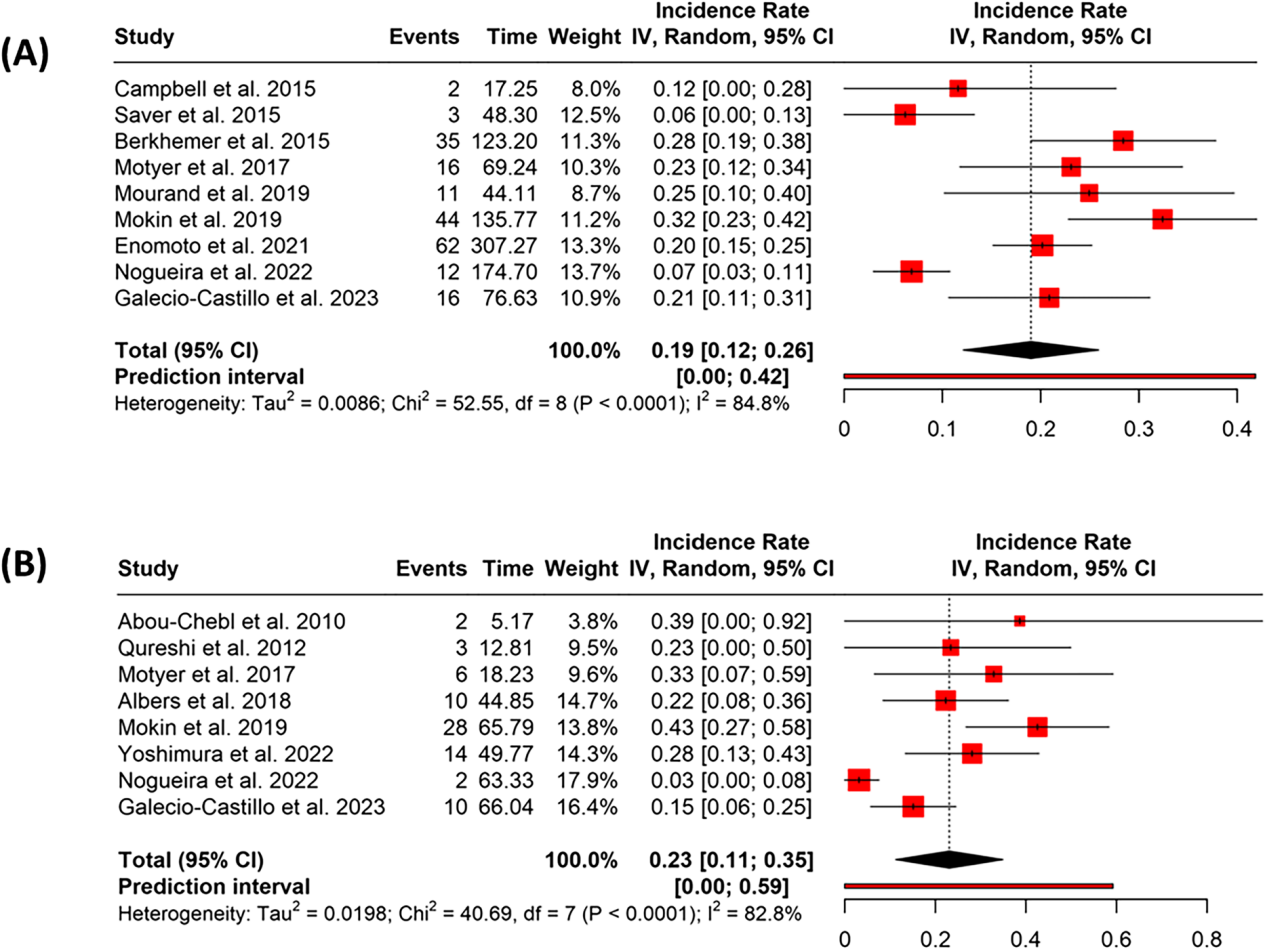
Forest plots showing the pooled incidence rates of symptomatic intracranial hemorrhage (sICH) following endovascular thrombectomy (EVT), stratified by treatment timing. **A** Studies reporting sICH incidence in patients treated within 6 h of symptom onset (early group). **B** Studies reporting sICH incidence in patients treated between > 6 and ≤ 24 h from symptom onset (late group). Pooled incidence rates were calculated using a random-effects model. Squares indicate study-specific incidence rates, with size proportional to study weight; horizontal lines represent 95% confidence intervals. Diamonds denote the pooled estimates. Substantial heterogeneity was observed in both groups (*I*^2^ > 80%)

**Fig. 3:**
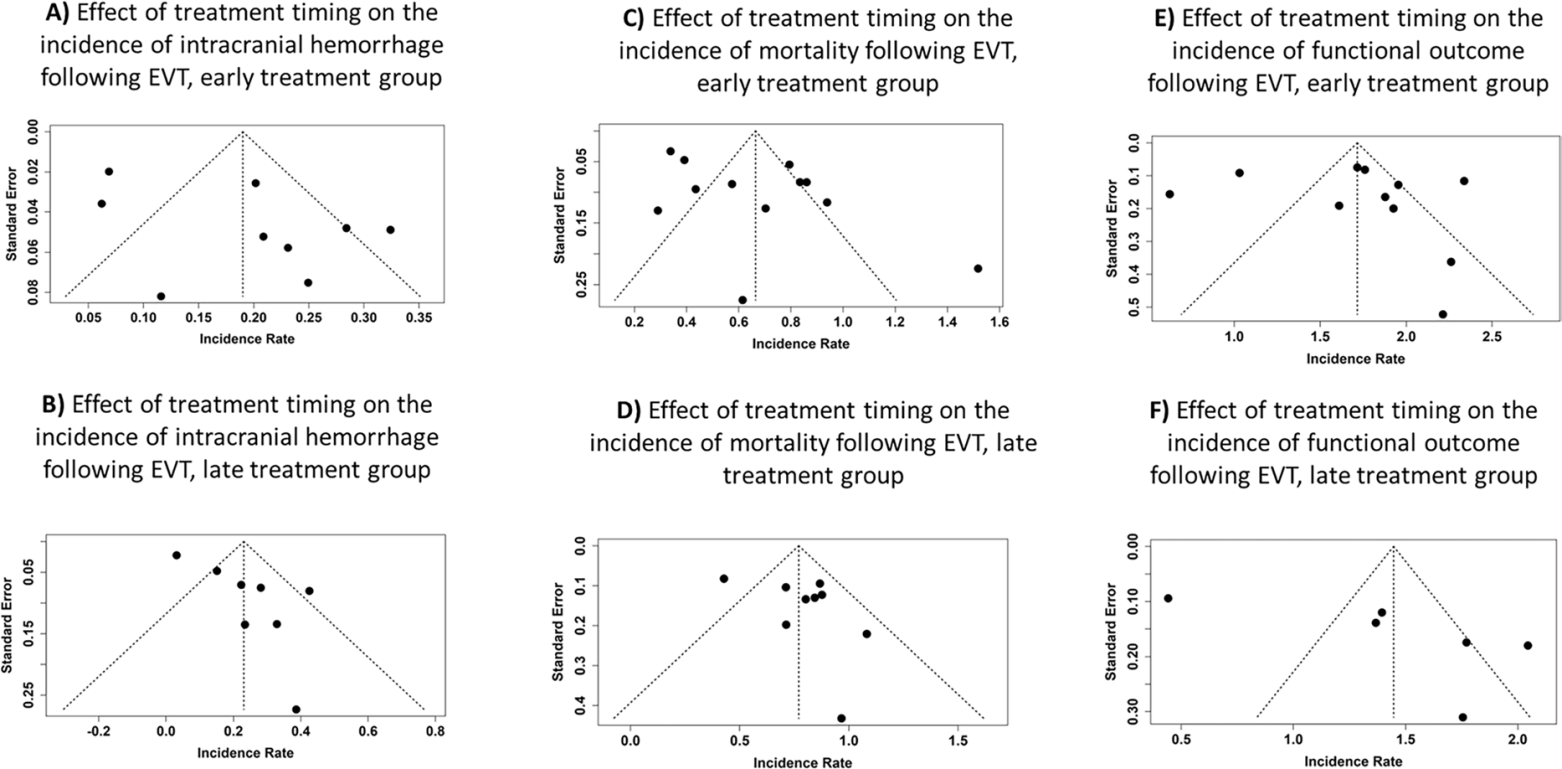
Funnel plots assessing potential publication bias in studies reporting symptomatic intracranial hemorrhage (sICH), mortality, and functional outcomes following endovascular thrombectomy (EVT), stratified by treatment timing. **A**, **B** Funnel plots for the incidence of sICH in the early (**A**) and late (**B**) treatment groups. **C**, **D** Funnel plots for mortality rates in the early (**C**) and late (**D**) treatment groups. **E**, **F** Funnel plots for functional outcome (modified Rankin Scale 0–2 at 90 days) in the early (**E**) and late (**F**) treatment groups. Each plot shows the incidence rate on the x-axis and standard error on the y-axis; the vertical dashed line indicates the pooled incidence rate, and the diagonal lines represent the pseudo 95% confidence limits. Visual asymmetry may suggest the presence of publication bias. Egger’s test results are described in the “Results” section

In the early treatment group (≤ 6 h), nine studies provided 201 events across 996.47 person-years. The pooled incidence rate was 0.19 (95% CI: 0.12–0.26), with substantial heterogeneity (*p* < 0.01, *I*^2^ = 85%) (Fig. 2A). The funnel plot did not suggest publication bias, which was consistent with Egger’s test (intercept = 3.18, 95% CI: − 0.85 to 7.21, *t* = 1.866, *p* = 0.104) (Fig. 3A).

In the late treatment group (> 6 h), eight studies contributed a total of 75 events over 325.99 person-years. The collective incidence rate was 0.23 (95% CI: 0.11–0.35), with significant heterogeneity (*p* < 0.01, *I*^2^ = 83%) (Fig. 2B). The funnel plot indicated potential publication bias, which was supported by Egger’s test (intercept = 3.07, 95% CI: 1.08–5.07, *t* = 3.768, *p* = 0.009) (Fig. 3B).

The incidence rate difference (IRD) between groups was − 0.028 (95% CI: − 0.0855 to 0.0288; *p* = 0.3307), and the incidence rate ratio (IRR) was 0.88 (95% CI: 0.6693 to 1.1589; *p* = 0.3314), indicating no statistically significant difference between groups.

### Mortality

**Fig. 4:**
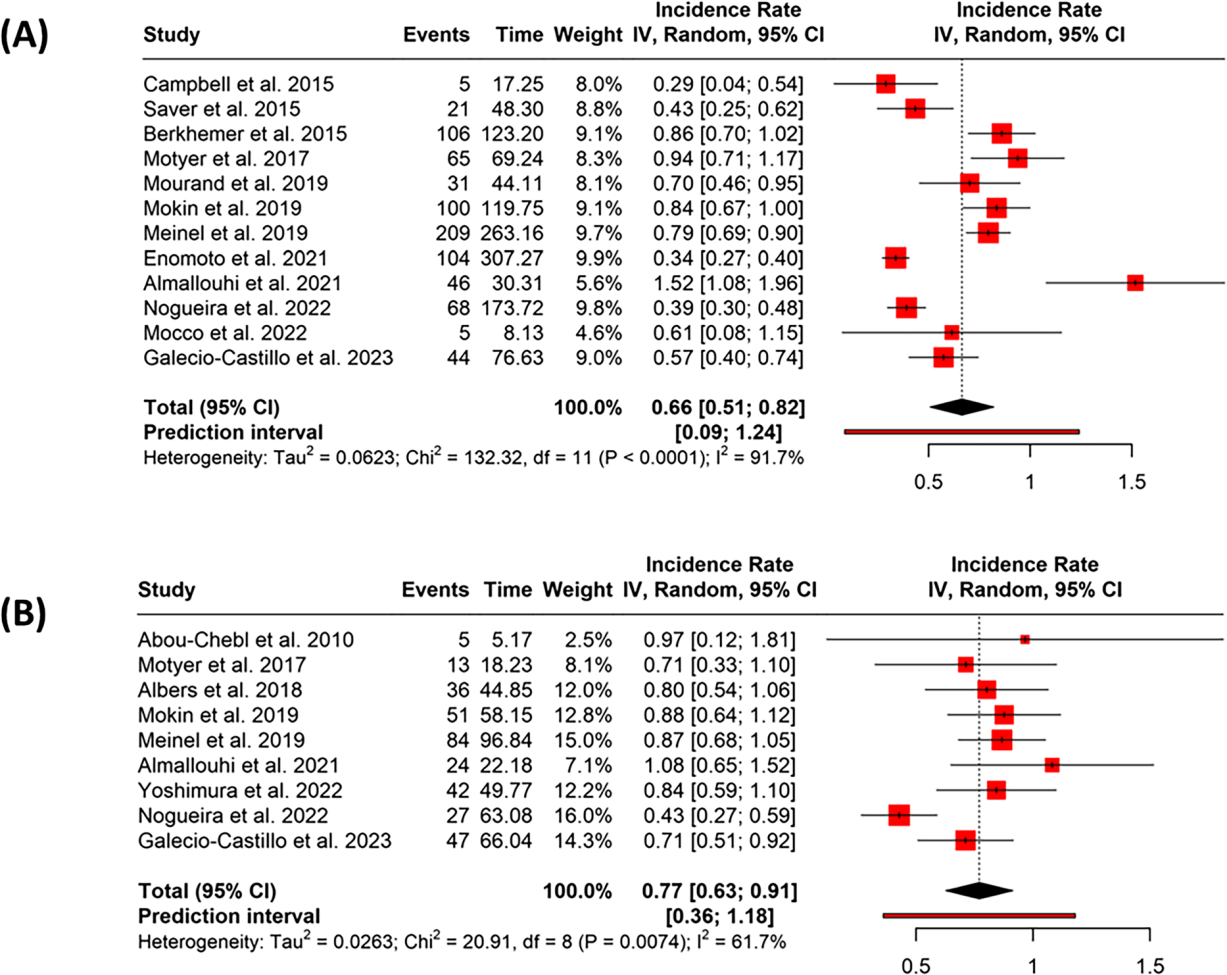
Forest plots showing the pooled incidence rates of 90-day mortality following endovascular thrombectomy (EVT), stratified by treatment timing. **A** Studies reporting mortality in patients treated within 6 h of symptom onset (early group). **B** Studies reporting mortality in patients treated between > 6 and ≤ 24 h from symptom onset (late group). Pooled incidence rates were estimated using a random-effects model. Squares represent individual study estimates with size proportional to their weight; horizontal lines indicate 95% confidence intervals. The diamond reflects the pooled estimate. Substantial heterogeneity was observed in the early treatment group (*I*^2^ = 91.7%) and moderate heterogeneity in the late treatment group (*I*^2^ = 61.7%)

In the early treatment group, 12 studies reported 804 events over 1281.0678 person-years. The pooled incidence rate was 0.66 (95% CI: 0.51–0.82), with high heterogeneity (*p* < 0.01, *I*^2^ = 92%) (Fig. 4A). The funnel plot indicated possible publication bias, supported by Egger’s test (intercept = 3.86, 95% CI: 0.11–7.61, *t* = 2.294, *p* = 0.045) (Fig. 3C).

In the late treatment group, nine studies reported 329 events across 424.3121 person-years. The collective incidence rate was 0.77 (95% CI: 0.63–0.91), with moderate heterogeneity (*p* = 0.01, *I*^2^ = 62%) (Fig. 4B). Funnel plot inspection did not suggest publication bias, and Egger’s test was not significant (intercept = 2.13, 95% CI: − 1.09 to 5.35, *t* = 1.564, *p* = 0.162) (Fig. 3D).

The difference between the two groups was statistically significant. The IRD was − 0.148 (95% CI: − 0.2373 to − 0.0583; *p* = 0.0012), and the IRR was 0.81 (95% CI: 0.7111 to 0.923; *p* = 0.0014), indicating a lower mortality rate in the early treatment group.

### Functional outcome (mRS)

**Fig. 5:**
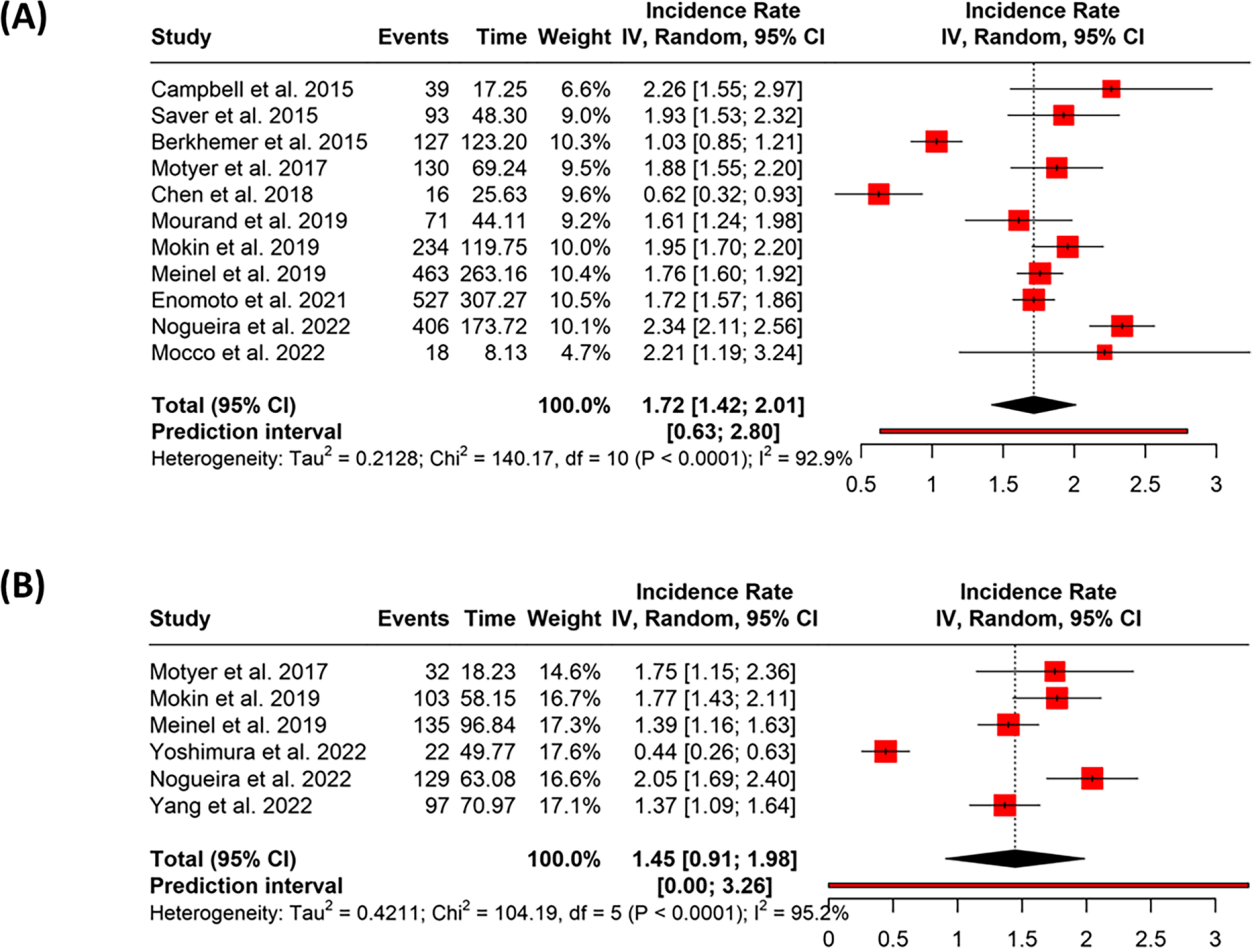
Forest plots showing the pooled incidence rates of favorable functional outcome (modified Rankin Scale score 0–2 at 90 days) following endovascular thrombectomy (EVT), stratified by treatment timing. **A** Studies reporting outcomes in patients treated within 6 h of symptom onset (early group). **B** Studies reporting outcomes in patients treated between > 6 and ≤ 24 h from symptom onset (late group). Pooled incidence rates were estimated using a random-effects model. Individual studies are shown as red squares with size proportional to their weight; horizontal lines indicate 95% confidence intervals. The diamond indicates the pooled estimate. Heterogeneity was high in both groups (*I*^2^ = 92.9% in early, *I*^2^ = 95.2% in late group)

In the early treatment group, 11 studies contributed 2124 events over 1199.7537 person-years. The pooled incidence rate was 1.72 (95% CI: 1.42–2.01), with very high heterogeneity (*p* < 0.01, *I*^2^ = 93%) (Fig. 5A). Funnel plot analysis did not reveal potential publication bias, and Egger’s test was not significant (intercept = 1.06, 95% CI: − 5.07 to 7.18, *t* = 0.39, *p* = 0.705) (Fig. 3E).

In the late treatment group, six studies contributed 518 events over 357.0431 person-years. The combined incidence rate was 1.45 (95% CI: 0.91–1.98), with considerable heterogeneity (*p* < 0.01, *I*^2^ = 95%) (Fig. 5B). The funnel plot showed no clear indication of publication bias, and Egger’s test did not suggest any asymmetry (intercept = 9.78, 95% CI: − 1.73 to 21.29, *t* = 2.359, *p* = 0.078) (Fig. 3F).

The difference between the groups was statistically significant. The IRD was 0.32 (95% CI: 0.1656 to 0.4735; *p* < 0.0001), and the IRR was 1.22 (95% CI: 1.108 to 1.3459; *p* < 0.0001), indicating a higher rate of favorable functional outcome in the early treatment group.

## Discussion

This systematic review and meta-analysis evaluated the impact of treatment timing on outcomes following EVT for acute ischemic stroke (AIS). Our findings support existing knowledge that earlier intervention (≤ 6 h from symptom onset) is associated with significantly lower mortality and higher likelihood of functional independence at 90 days, without an increased risk of symptomatic intracranial hemorrhage (sICH). These results reinforce the principle that “time is brain” [47] and emphasize the critical importance of minimizing delays in initiating EVT. As previously estimated, the typical patient with untreated stroke loses approximately 1.9 million neurons per minute, highlighting the devastating biological cost of delayed reperfusion [47].

The observed 19% relative reduction in mortality (IRR 0.81) and 22% increase in favorable functional outcomes (IRR 1.22) with early EVT align with prior registry data and trial-level meta-analyses showing a steep time–benefit gradient for thrombectomy [23, 24]. Importantly, our pooled estimates reflect real-world practice, incorporating both randomized controlled trials and well-conducted observational studies. Concerns about procedural risk, particularly sICH, have largely been alleviated for early EVT, following the pivotal trials that established its safety and efficacy within the 6-h window. However, in the late treatment window, the risk of sICH remains a critical consideration, as reperfusion of necrotic or structurally compromised tissue may increase the likelihood of hemorrhagic transformation. Notably, our analysis did not demonstrate a statistically significant increase in sICH in the late group compared to early intervention, which may reflect careful patient selection in these studies. These findings underscore the importance of refining methods to identify slow progressors who maintain salvageable tissue and can safely benefit from EVT beyond 6 h.

Our results also shed light on the nuanced benefits of delayed EVT. Although outcomes were significantly better with early intervention, the late window group still demonstrated nontrivial rates of functional independence, affirming the value of EVT beyond 6 h in appropriately selected patients. This supports existing guidelines that recommend advanced imaging to identify salvageable brain tissue in the extended time window [48]. Nonetheless, the wide heterogeneity in outcomes among late-window studies highlights variability in patient selection criteria, imaging protocols, and institutional expertise—factors that likely contribute to the so-called late window paradox [14, 15, 21].

Several studies have reported favorable outcomes in select late-presenting patients, often due to favorable collateral circulation, slow infarct progression, or imaging-based selection [49–53]. However, such findings should not be misinterpreted as suggesting that later EVT is equivalent to earlier. In clinical practice, intentional delays in EVT do not occur [6], as strict quality metrics, including door-to-groin puncture time, are closely monitored by accrediting bodies such as Joint Commission for Accreditation of Comprehensive Stroke Center. Delays are most often attributable to patients arriving late to thrombectomy-capable centers rather than hesitancy to initiate treatment. These observations highlight the need for individualized patient assessment and underscore that the benefit of late EVT is not uniformly applicable across all AIS cases. Future research aimed at better characterizing the biological and vascular determinants of favorable collaterals and slow infarct progression—such as genetic, metabolic, or age-related factors—may help refine patient selection. This line of investigation could also inform the development of adjunctive therapies, including agents targeting age-related cellular mechanisms or metabolic pathways, aimed at enhancing cerebrovascular resilience and potentially extending the window of opportunity for effective reperfusion. From a systems-level perspective, the capacity to deliver EVT beyond 6 h depends on timely access to stroke centers with the necessary infrastructure [6]. Fortunately, requirements for Comprehensive Stroke Center certification ensure that necessary resources, such as 24/7 advanced imaging and EVT capability, are in place to support timely treatment within and beyond the conventional 6-h window when clinically appropriate.

Aging is the primary risk factor for ischemic stroke [1–3] and critically shapes both vulnerability to cerebrovascular events and capacity for recovery. From a geroscience perspective, age-related deterioration of both macrovascular and microvascular health [54] and cerebrovascular reserve [55] contributes to impaired ischemic tolerance and reduced stress resilience in older adults. This biological backdrop may partly explain the observed between-individual variability in functional outcomes following EVT—particularly in the late window, where patient selection depends heavily on the persistence of viable tissue and the capacity for neurovascular recovery [26]. Notably, inter-individual differences in biological aging [56–61] may supersede chronological age in determining EVT benefit. Some older patients may exhibit relatively preserved neurovascular resilience and cerebrovascular collaterals, while others experience accelerated cerebrovascular aging due to comorbidities such as hypertension [62, 63], diabetes [64–66], or clonal hematopoiesis of indeterminate potential (CHIP) [67]. Integrating geroscience-informed biomarkers—such as frailty indices [68], epigenetic [69] and proteomic clocks [56, 70, 71], neuroimaging markers of small vessel disease [72, 73], AI-based brain age imaging biomarkers [74–76]—into future EVT trials may allow for improved patient stratification and more personalized treatment windows. Ultimately, understanding how the hallmarks of aging [77] influence cerebrovascular health [54, 55], repair and reperfusion response could pave the way for interventions that extend the benefit of EVT to a broader aging population. Looking ahead, translational geroscience interventions aimed at enhancing resilience and improving cerebral microcirculation—such as senolytic therapies, NAD⁺ boosters, or other metabolic modulators [78–81]—hold promise in extending the therapeutic window and improving stroke outcomes in older adults.

This study has several strengths, including comprehensive literature coverage, stratified quantitative synthesis, and inclusion of clinically relevant endpoints. However, several limitations must be acknowledged. First, heterogeneity across studies was moderate to high, reflecting variation in patient populations, imaging criteria, and procedural techniques. We could not meta-analyze the effects of comorbidities, prior stroke, or frailty status due to inconsistent reporting across studies. These factors likely contribute to inter-individual variability in EVT benefit and warrant further investigation. Second, despite robust statistical methods, residual confounding is possible, particularly in observational studies. Third, we did not assess outcomes in strokes beyond 24 h from last known well, where emerging evidence suggests further benefit in select cases [82]. Lastly, we would like to note that functional outcomes in very old patients (e.g., > 85 years) remain understudied in the delayed EVT window, and biologically informed stratification (e.g., frailty or biological age) may be more predictive than age cutoffs.

In conclusion, early EVT (within 6 h) offers significant clinical advantages in terms of survival and functional recovery. These findings support continued efforts to streamline acute stroke workflows and reduce door-to-reperfusion times. While early EVT remains the gold standard for patients with acute large vessel occlusion, our findings suggest that carefully selected late-window patients—particularly slow progressors identified by advanced imaging—can still achieve favorable outcomes without excess hemorrhagic risk. Future research should explore molecular approaches to optimize selection criteria for extending the thrombectomy window and guiding personalized stroke care.
